# Supplementary material for: Complex‐centric proteome profiling by SEC‐SWATH‐MS
Source: Mol Syst Biol. 2019 Jan 14;15(1):e8438. doi: 10.15252/msb.20188438 (PMC6346213; doi:10.15252/msb.20188438)
Supplement: Supplementary file 7 — Dataset EV6 [file MSB-15-e8438-s007.zip › feature_plots_bioplex/Q9Y275.pdf]

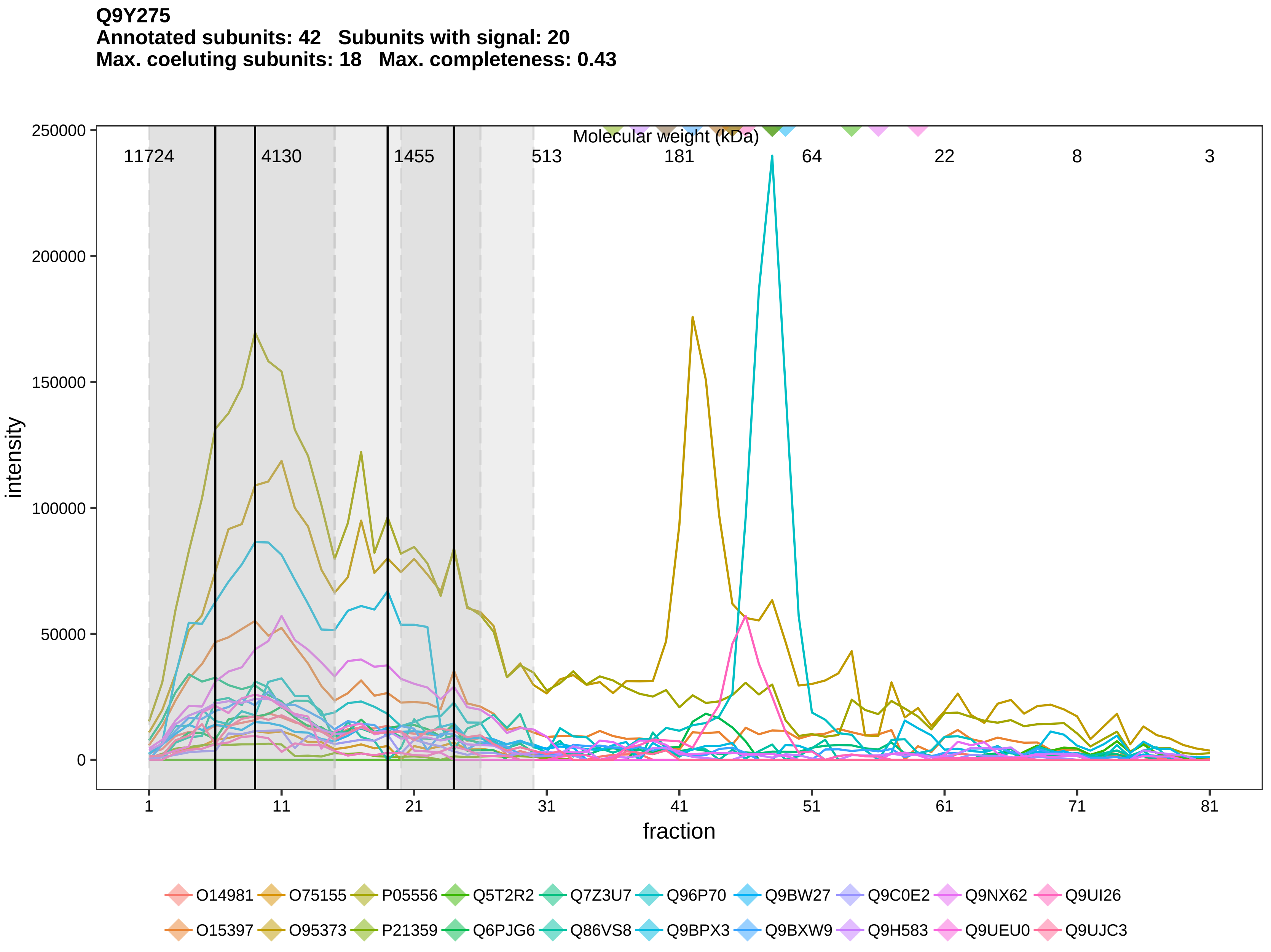

Q9Y275
Annotated subunits: 42 Subunits with signal: 20
Max. coeluting subunits: 18 Max. completeness: 0.43
Molecular weight (kDa)
11724
4130
1455
513
181
64
22
8
3
intensity
250000
200000
150000
100000
50000
0
1
11
21
31
41
51
61
71
81
fraction
O14981
O75155
P05556
Q5T2R2
Q7Z3U7
Q96P70
Q9BW27
Q9C0E2
Q9NX62
Q9UI26
O15397
O95373
P21359
Q6PJG6
Q86VS8
Q9BPX3
Q9BXW9
Q9H583
Q9UEU0
Q9UJC3
